# Freestanding photocatalytic materials based on 3D graphene and polyporphyrins

**DOI:** 10.1038/s41598-018-23345-y

**Published:** 2018-03-22

**Authors:** Martina Ussia, Elena Bruno, Emanuela Spina, Daniele Vitalini, Giovanna Pellegrino, Francesco Ruffino, Vittorio Privitera, Sabrina C. Carroccio

**Affiliations:** 1 Department of Physics and Astronomy, University of Catania, via Santa Sofia 64, 95123 Catania, Italy; 2 CNR-IMM, Via Santa Sofia 64, 95123 Catania, Italy; 3 CNR-IPCB, Via Paolo Gaifami 18, 95126 Catania, Italy

## Abstract

A new concept in the formulation of hybrid nanostructured materials combining high quality graphene 3D supported by Nickel foam and polyporphyrins for visible light photocatalytic application is here reported. Our innovative approach involves the development of a freestanding device able to: i) offer a high surface area to bind the photosensitizers by π-π interactions, and ii) enhance stability and photocatalytic efficiency by using cyclic porphyrin polymers. For these purposes, homo- and co-polymerization reactions by using different porphyrin (free or zinc complexed) monomers were performed. The microscopic structures and morphology of graphene polymer nanocomposites were investigated by using Scanning Electron Microscopy (SEM), X-ray photoelectron spectroscopy (XPS) and Atomic Force Microscopy (AFM). Finally, photocatalytic activity under visible light irradiation of the obtained nanocomposites was tested, by using methylene blue (MB) as organic pollutant. The obtained data suggested that hindered cyclic polymeric structures stacked on graphene surface by non-covalent interactions, restrict the formation of non photoactive aggregates and, as a consequence, induce an enhancement of photocatalytic activity. Remarkably, our systems show a degradation efficiency in the visible-light range much higher than other similar devices containing nanoporphyrin units reported in literature.

## Introduction

The design of graphene-based hybrid nanomaterials for photocatalytic applications is one of the most promising routes in the field of water remediation^1–3^. Indeed, the combination of graphene with photoactive materials aims to strongly improve the photocatalytic performance of the formulated systems by boosting the separation and transfer of photo-generated charges, which constitutes a critical step in photocatalytic reactions. As it turns out, the merging of the excellent visible-light absorption abilities of organic photosensitizers with the charge transfer properties of graphene-based materials (graphene, graphene oxide and reduced graphene oxide) represents the cutting-edge on water treatment technology^4^. Moreover, the low cost, eco-friendliness, flexibility and versatility of the molecular design of these basic organic molecules constitute appealing features, both environmentally and economically.

Among these, porphyrin molecules have shown great potential in visible-light photocatalytic applications because of their large extinction molar coefficient in the visible-light region and the well-defined organic structures with controllable size and morphology^5^. As thoroughly reported in the literature, the chemical structures of porphyrins, as well as the sizes and morphologies of their self-assembly aggregates, negatively influence their photocatalytic efficiency^6–8^, being limited by the fast recombination of photo-induced electron-hole pairs and the low photo-stability of porphyrins themselves. Indeed, the latters tend to undergo photobleaching. In spite of these limitations, the self-assembly of nano-porphyrins have been recently proven to explicate great potential in visible-light photocatalytic applications, promoting a boost of dye degradation efficiencies^5^. As reported by Guo *et al*., differences in molecular packing lead to morphology-dependent catalytic performance. In particular, a distinct photocatalytic performance is observed whenever a fibrous zinc-tetra(4-pyridyl)porphyrin nanostructure is used with respect to the spherical one in the degradation of organic pollutants^6^. Similar findings have been reported for nanostructured meso tetra(4-carboxyphenyl)-porphyrins (TCPP), suggesting that porphyrins molecules existing in a total J-aggregation facilitate the electron transfer process that prolongs the life-time of the excited state and, therefore, the photocatalytic performance^9^.

Significant efforts in electron transfer process have been made also by anchoring porphyrins onto graphene based materials via covalent and non-covalent interactions (e.g., π-π stacking or van der Waals and/or electrostatic interactions) strategies, which permit to bind molecules to the selected substrate^10–17^. Some complications arise from the use of graphene-based materials as co-catalysts. In fact, although these materials generate the benefits discussed above, some tricky features have to be taken into account during the photodegradation processes, such as the “shielding effect” as well as the radical scavenger activity. The first phenomenon is induced by the high weight addition of graphene-based materials as co-catalyst. Due to their opacity, a weakening of the light irradiation depth through the graphene-based composites restricts the efficiency of graphene in promoting the photoactivity^18^. This intrinsic negative effect imposes a weight addition ratio of graphene derivatives into the composite lower than 5%. However, greater amounts of carbon materials would be necessary to inhibit the recombination of electron-hole pairs photogenerated by the photocatalyst^18^. In covalently graphene oxide (GO) and reduced graphene oxide (rGO) functionalized composites, an increasing number of photoactive sites are clearly related to an improving of photocatalytic efficiencies. To boost the number of such sites, it is indispensable to increase the weight addition ratio or, alternately, the oxidation points onto the graphene surface. The latter indeed introduces significant structural defects that strongly affect the electron mobility and thus the charge transfer and separation processes. Furthermore, a huge adsorption capacity against organic contaminants in water is displayed from oxidised graphene-based materials. This peculiar property, that in the case of GO versus methylene blue (MB) was quantified at 714 mg/g^19^, makes difficult to discern between adsorption and photodegradation processes during photocatalytic tests. Finally, another crucial point is related to the antioxidant activity of carbon materials versus organic molecules. Multiple experiments have shown that graphene-based materials are an effective scavenger of OH^•^ radicals, avoiding the desired purification catalyzed by photoactive molecules^20^.

To obtain an efficient device overcoming the aforementioned disadvantages, a freestanding hybrid nanocomposite by using high quality graphene 3D supported by Nickel foam and photoactive macromolecules was realized. The novelty of our approach consists in exploiting extensive non-covalent interactions between the high surface area of graphene co-catalyst and a coating of cyclic porphyrin polymers. The unusual choice of porphyrin polymers (PPrs) as photoactive sites is based on two assumptions: i) their hindering conformational geometries might significantly avoid formation of agglomerates of single porphyrin units, that act the rapid quenching of excited state; ii) if compared to isolate photo-active molecules, polymers are able to much easily coat and protect the co-catalyst surface. Moreover, our structure shows very high avalaible surface thanks to its foam nature, further improving its photocatalytic performances.

This new concept of graphene polymer nanocomposites formulation might avoid the shielding effect as well as the radical scavenger activity, maximising the charge transfer process during photocatalysis. The photocatalytic efficiencies of these nanocomposites were tested and compared to graphene/porphyrin monomers by photodegrading MB dye in water under visible-light irradiation. Our results are below reported and discussed.

## Results and Discussion

### Synthesis of graphene foam (GF) and characterization

**Figure 1:**
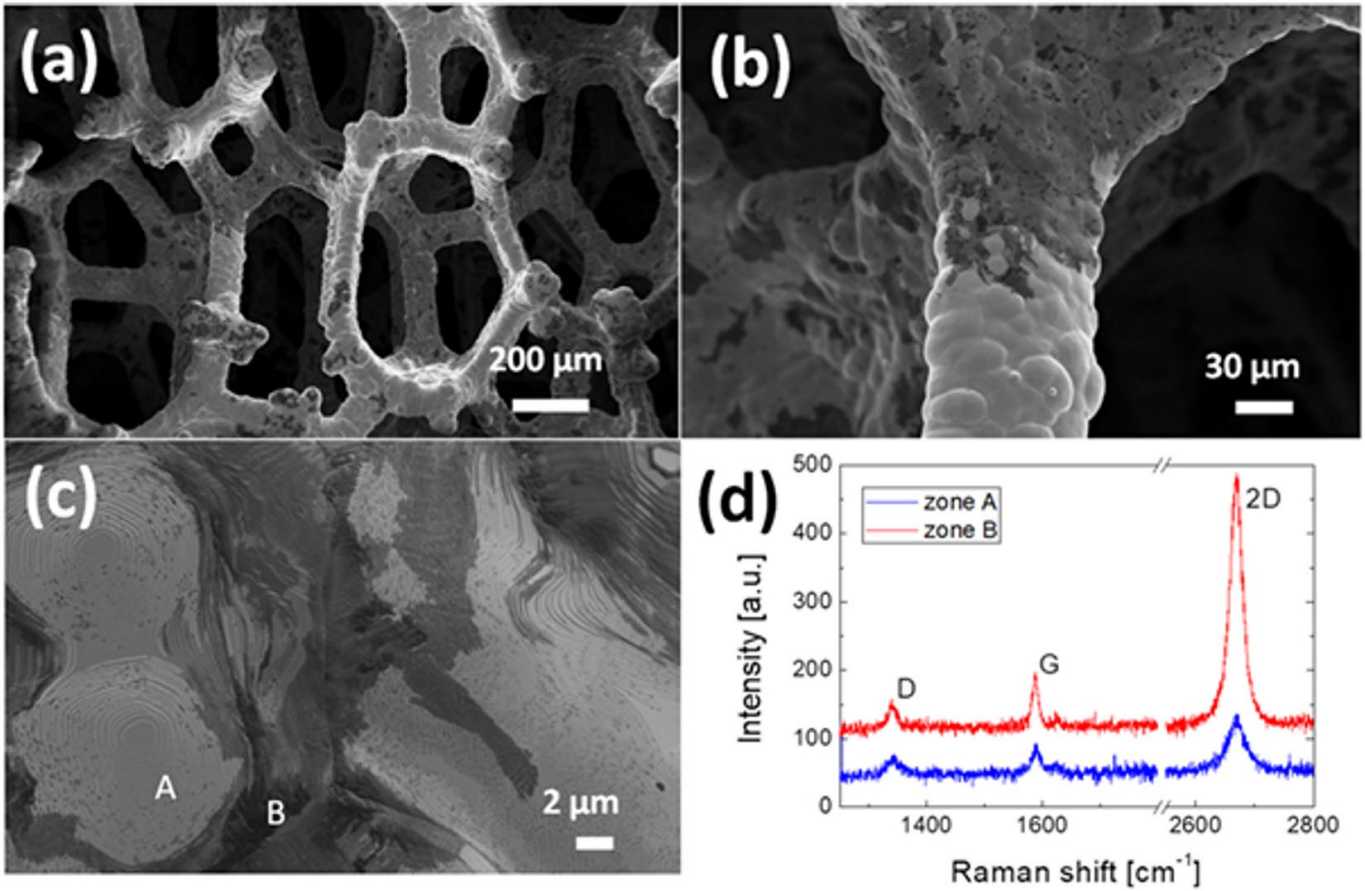
(**a**–**c**): SEM images obtained in the Inlens mode of the nickel-graphene foam at different magnifications; (**d**): Raman spectra obtained on different areas of the foam with different grey contrast (e.g. zone A and B of panel (**c**)).

The nickel-graphene foam obtained by CVD growth is shown in Fig. 1. The structure was analyzed by SEM at progressive magnifications [Fig. 1 (a–c)]. It is evident that the graphene film adheres to the surface of the nickel foam, reproducing its morphology, but it shows a non-homogeneous distribution, as evidenced by the different grey contrasts over the sample. Nonetheless, this contrast does not mean that the nickel is not fully covered, but it is only due to a different quality of the graphene film. This is confirmed by Raman spectra [Fig. 1 (d)] that evidence the typical G and 2D peaks of either in the brighter and darker regions. The irregular distribution of graphene (going from monolayer to multilayers) is well known on nickel substrates, due to the graphene growth process occurring by carbon segregation and precipitation^21^. Indeed, this growth mechanism is not self-limiting like on copper.

### Synthesis and characterization of homo-polyporphyrin (homo-PPr) and co-polyporphyrin (co-PPr)

**Figure 2:**
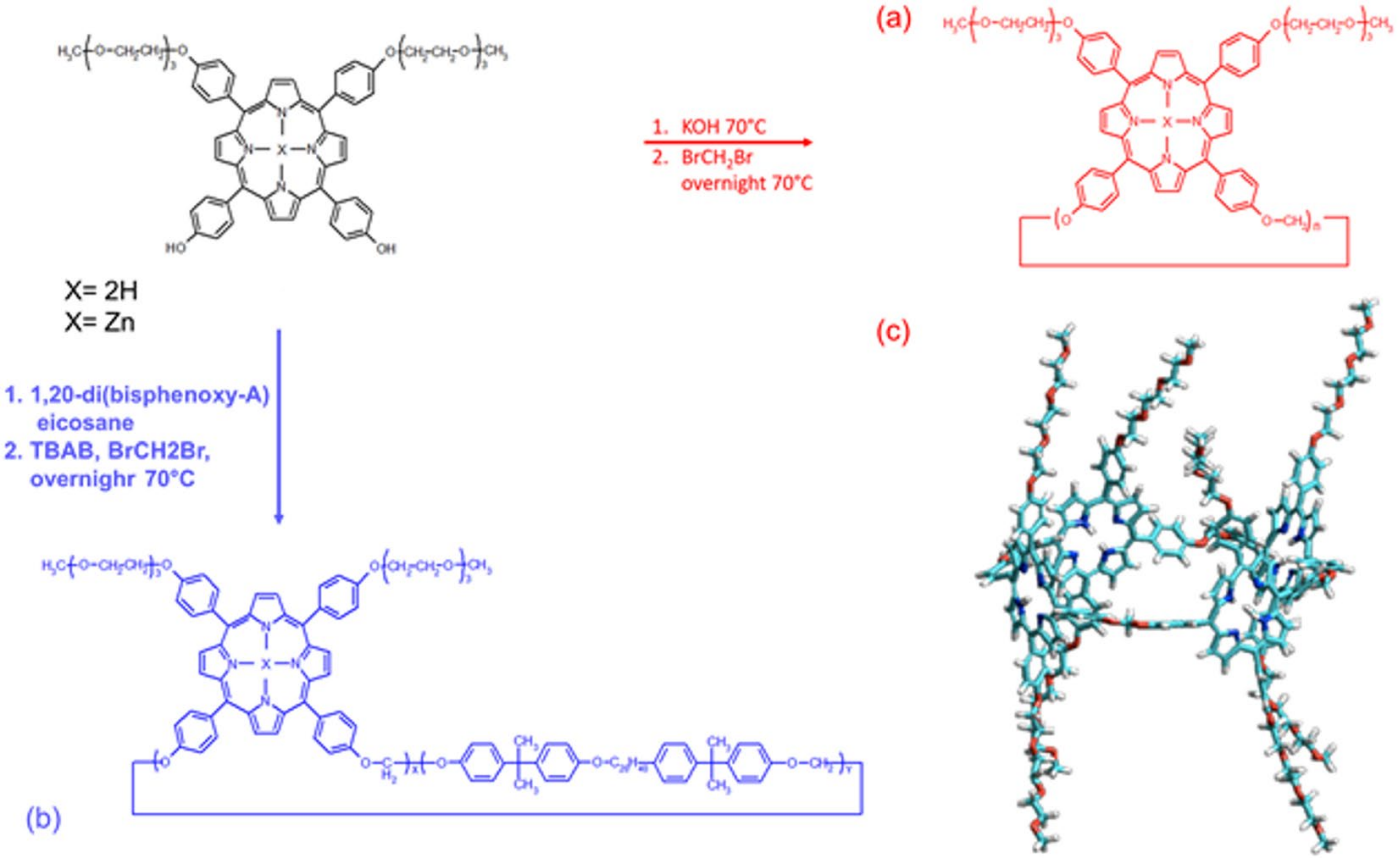
Preparation of (**a**) homo-PPrs (red line) and (**b**) co-PPrs (blue line). Molecular model of cyclic tetramer of Poly trans-Prs (as example of homo cyclic-porphyrin polymers conformational structure, reproduced from ref.^23^ with kind permission of Elsevier) (**c**).

The heterogeneous catalysis from porphyrins and metal-porphyrins under visible-light irradiation is well reported in the literature^4^. Porphyrin systems can act as photocatalytic media as free molecules, supported molecules, nanostructured assemblies and thin films^4,6,10,14^. Whatever is the strategy adopted, the formation of porphyrin agglomerates triggers the rapid quenching of excited state preventing photocatalysis. We used a simple polymerization process to concatenate higher number of photoactive sites in more stable cyclic chain structures^22–25^. In addition, cyclic polymerization of such porphyrins were chosen to restrain the formation of non-photoactive porphyrin aggregates^26^. In Fig. 2 (a,b) were depicted the synthetic pathways of polymerization for homo-PPr [Fig. 2 (a) red line] and co-PPr [Fig. 2 (b) blue line]. We assume that the GF assemblies with conformational hindered PPrs cycles, having also two hampering pendant groups in 5 and 10 positions for each molecule [see Fig. 2 (c)], might negative affect in coordinating other similar molecules. In the first polymerization the reactions were also carried out by using porphyrin monomer having zinc as metal center, hoping to find and discriminate, eventually, differences in photochemical behaviors of GF/PPrs devices. In particular, we have selected Zn porphyrin derivative, because of the considerable interest in photocatalytic application^8,13,27^. The synthetic details of polymerization reactions of homo-PPr and co-PPr are reported in the experimental section. The obtained materials were purified and characterized by MALDI-TOF MS, GPC and UV-Vis spectroscopy. MALDI-TOF analysis, performed after samples purification, indicated that zinc derivate (homo-PPr Zn) contained oligomers lower than 3 repeat units, whereas polymerization of porphyrin Zinc free (homo-PPr), displayed a higher number of porphyrin units along the cyclic chains (see Fig. 1s ESI^†^). The Table 1 reports the molar masse (MM) values of homo-PPr and co-PPr calculated by using GPC analysis. Data reported reveal higher MM values for co-PPr if compared with homo-PPr sample. A total content of 37% of porphyrin into the co-PPr was estimated by using UV-Vis method (Table 1).

**Table 1:** Composition, yield and Molar Masses of synthesized polymers.

Polymer	Nominal % Porphyrin molar content	Actual % Porphyrin molar content	Polymer yield (w/w)^a^	Mw^b^	Mn^b^
**Homo-PPr**	100	100	80%	4932	3808
**Co-PPr**	50	37,5	75%	10586	9241

^a^Percent of polymeric material with respect to the total amount of starting monomers.

^b^Molar masses values calculated by using PMMA as GPC standards.

### Formulation of freestanding GF/polymer nanocomposites

**Figure 3:**
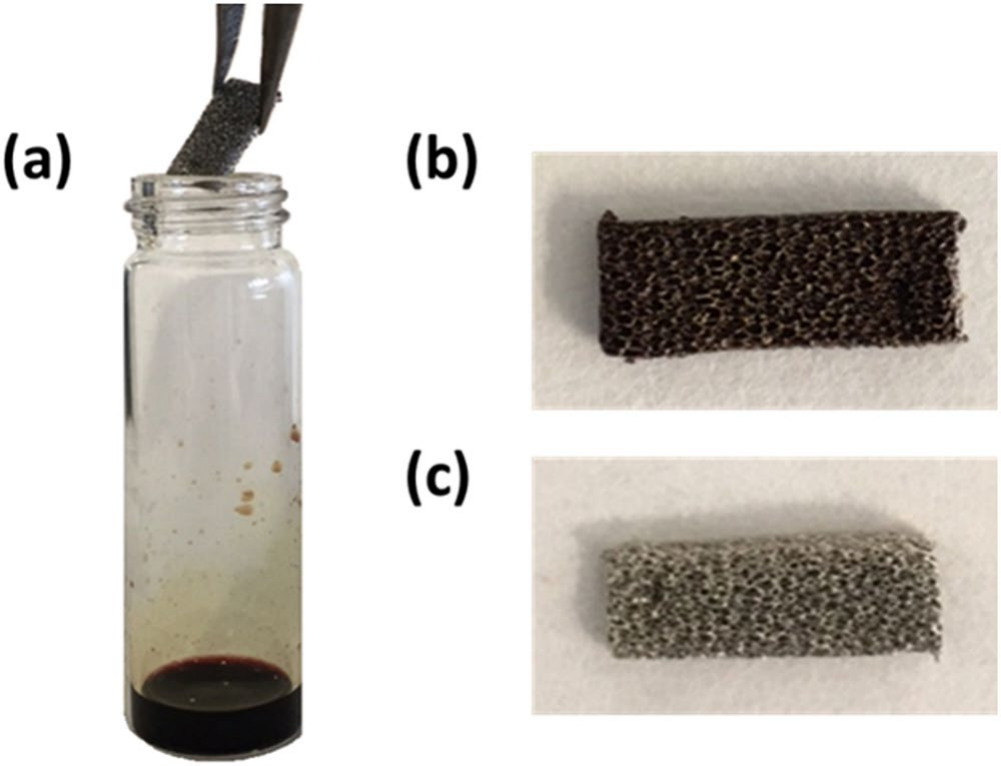
Formulation of GF/polymer nanocomposites: (**a**) the immersion of GF in PPrs solutions, (**b**) dried GF/homo-PPr and (**c**) dried GF/co-PPr samples.

To take advantage in terms of photocatalytic efficiencies, we formulated a robust freestanding device able to maximize the π-π and electrostatic interactions between GF and photoactive molecules, combining them via a simple deposition method. At the same time, problems deriving from the use of graphene materials wanted to be circumvented. For these purposes, nickel foam covered by graphene, with a surface area of 850 m^2^/g, was used as solid support and embedded with the polymeric photosensitizers. The GF pieces of about 5 mm × 20 mm were characterized by Scanning Electron Microscopy (SEM) and Raman spectroscopy (Fig. 1), and weighted. The GF/cyclic porphyrin polymers were formulated by dissolving known amounts (~10 mg) of each polymer sample (homo-PPrs and co-PPr) in 1 mL of DMF. After, the GF pieces were immersed into the obtained solutions overnight, removed from the vials and dried under vacuum overnight at 50 °C [Fig. 3 (a–c)]. The obtained materials were weighted again to estimate the amount of cyclic porphyrin polymers non-covalently attached to the GF surface. In both case (homo- and co-PPr), the weights registered after deposition indicated amounts lesser than 0,4 mg. To gain information on the chemical structure of the two assembled materials, we washed them several times by using DMF and CHCl_3_. After, XPS analyses were performed.

**Figure 4:**
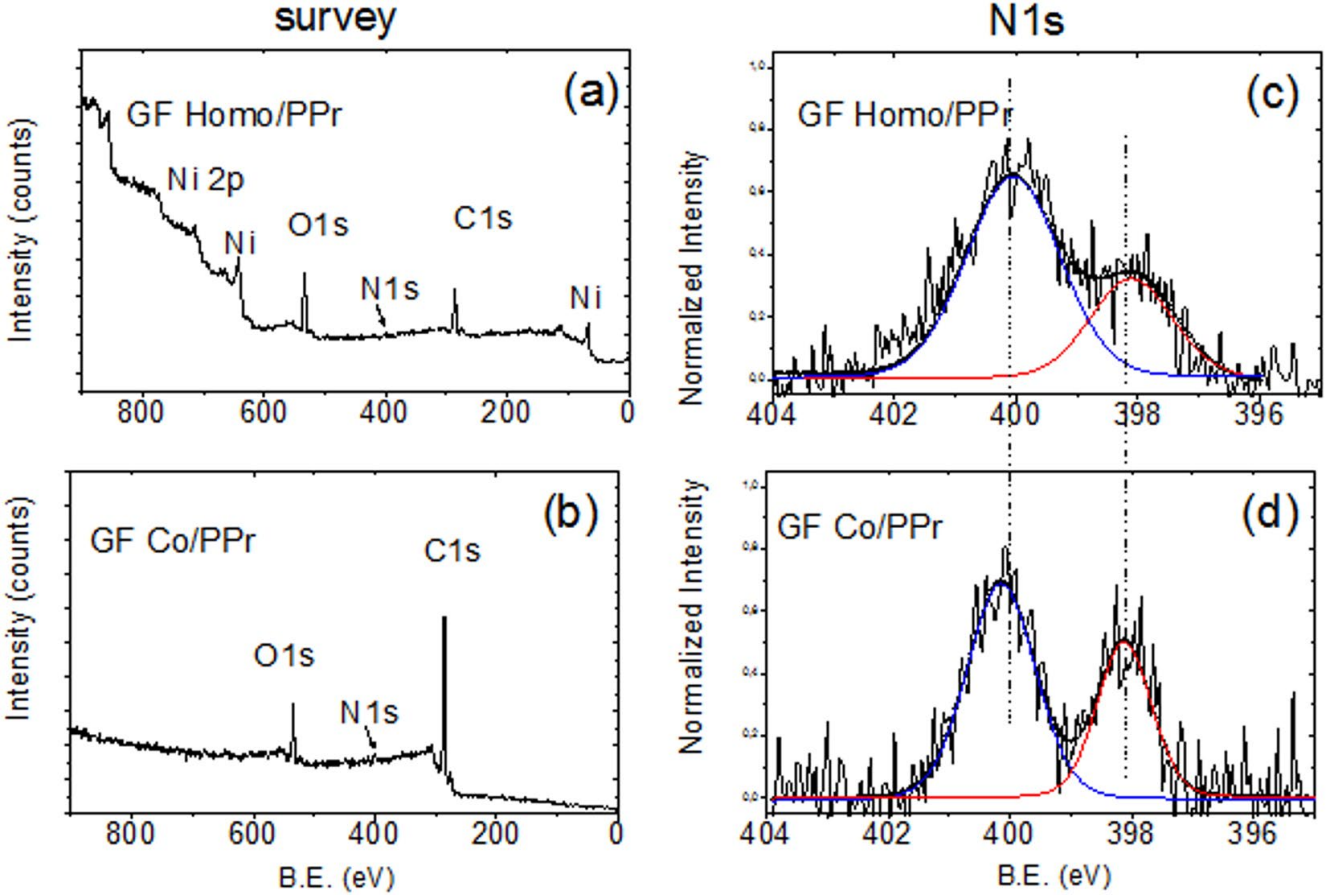
Left panel: wide range XPS spectra (survey) of (**a**) GF Homo-PPr and (**b**) GF Co-PPr; Right panel: high resolution N1s XPS spectra of (**c**) GF Homo-PPr and (**d**) GF Co-PPr.

Data on the surface composition of both the porphyrin-based freestanding devices were collected. The wide range XPS spectra (survey) related to the GF/homo-PPr and GF/co-PPr composites are shown in the left panel of Fig. 4. As visible, both the survey spectra evidence the presence of carbon and oxygen species, as expected. The nitrogen contribution, instead, is not visible because of the low amount in both the nanocomposites (see Table 2). The spectral signals from the nickel substrate are visible just in GF/homo-PPr sample, thus indicating that in the case of the co-polymer, a smoother coating is produced on the Ni-graphene surface than in the homo-polymer. The elemental percentages reported in Table 2 show that the amount of carbon is significantly higher in the case of GF/co-PPr than in GF/homo-PPr. This finding is in agreement with the molecular structures of the two compounds sketched in Fig. 2, with the copolymer showing long organic spacers between the porphyrin units.

**Table 2:** XPS relative atomic percentages as obtained by the spectra of GF Homo-PPr and GF Co-PPr

	C 1s (%)	O 1s (%)	N 1s (%)	Ni 2p
**GF Homo-PPr**	57.8	30.5	2.7	9.0
**GF Co-PPr**	86.3	11.2	2.0	<0.5

In order to inspect the electronic structure of the porphyrin-based compounds, we investigated the high-resolution region related to the N1s and C1s signals^28,29^ (see Fig. 2s ESI^†^ for C1s details). As widely reported in the literature, the N1s XPS represents a sensitive probe of the charge distribution in the porphyrin macrocycle, being strongly affected by their periphery and intermolecular interactions as well^30–34^. In agreement with the literature data related to the free-base porphyrin, the N1s signal consists of a doublet with the two components centered at 400.2 eV and at 398.2 eV, in both GF/Co-PPr and GF/Homo-PPr spectra. Such binding energy (BE) values are diagnostically attributed to the presence of the pyrrolic (−NH−) and to the iminic (−N = ) species, characteristic of the porphyrin ring^35,36^. Nonetheless, as seen by comparing Fig. 4 (c,d), the bandwidths of the pyrrolic and iminic components are notably different in both cases, being the Full Width at Half Maximum (FWHM) ~1.34 eV in the case of GF/co-PPr and ~1.82 eV in that of GF/homo-PPr. Such dissimilarity is attributed to a diverse chemical surrounding experienced by the nitrogen atoms of the macrocycle in the two porphyrin-based compounds. In particular, we evaluate that the enlargement of the bands observed in the case of GF/homo-PPr can be caused by the intermolecular stacking occurring between the porphyrin unit along the chains. On the other hand, in the case of GF/co-PPr, such intermolecular interaction is limited by the long organic sequences of aromatic/aliphatic spacers that separate the porphyrin units in the co-polymer. These observations are confirmed by the XPS analyses carried out on the powders of homo-PPr and co-PPr, taken as references (see Fig. 2s. ESI^†^). The analyses, in fact, further demonstrate the spectral differences between the two porphyrin-based structures, showing the broadening of the N1s XPS bands in the case of homopolymers, compared with the copolymers. Based on our results, we retain that the organic chains used as linker to join the porphyrinic units can modulate the intermolecular π-π stacking of the aromatic rings and, consequently, the final electronic structures of the macromolecular system.

**Figure 5:**
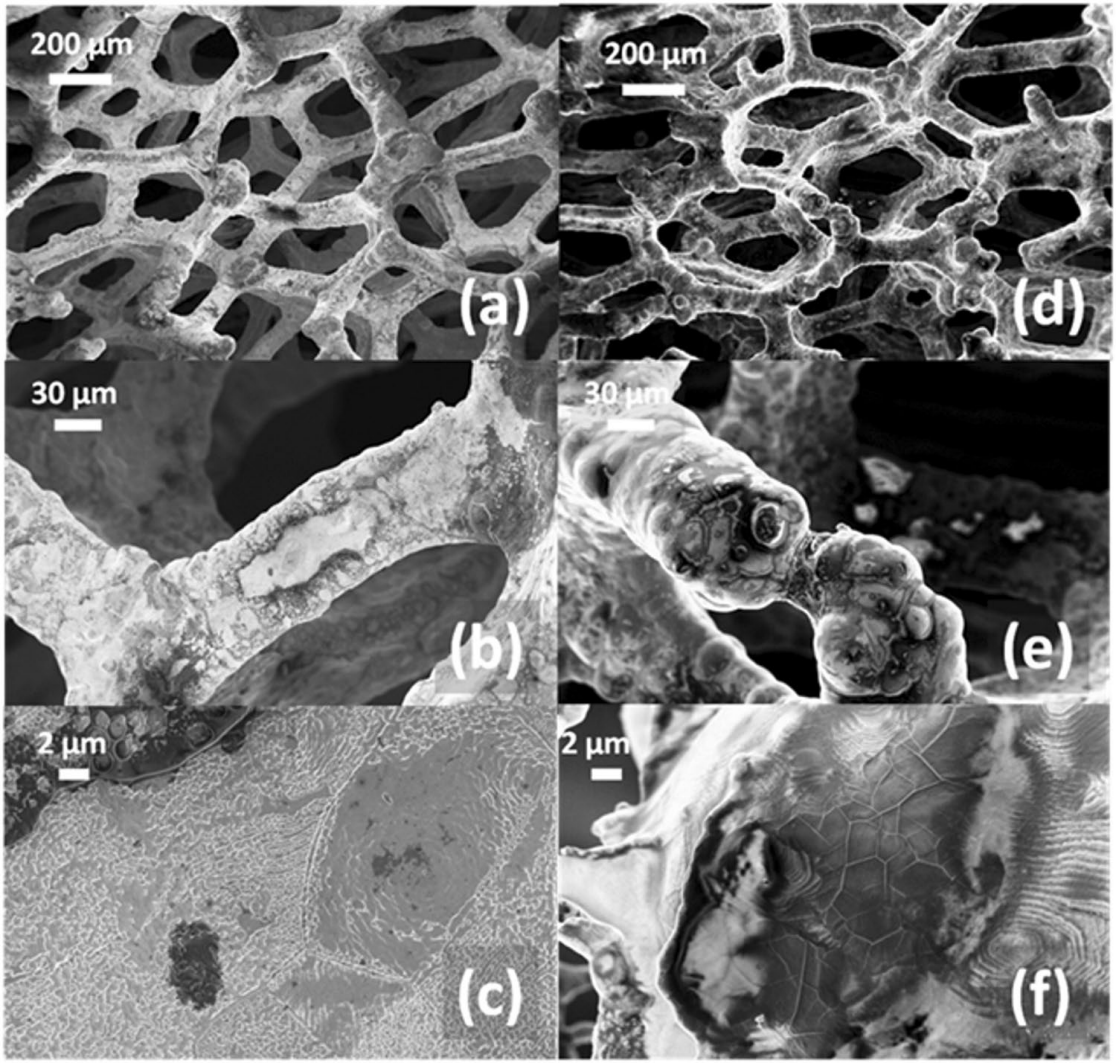
SEM images obtained in the Inlens mode at different magnifications of: the as-grown nickel-graphene structure covered with the homo-PPrs (**a**–**c**) and with the co-PPr films (**d**–**f**).

SEM analysis of GF/homo-PPr and GF/co-PPr were also carried out to observe the morphology of polymeric coating onto the GF surface. In Fig. 5, the different abilities in covering the nickel-graphene foam for the homopolymer [Fig. 5 (a–c)] and the copolymer films [Fig. 5 (d–f)] were reported and compared. In both cases, the polymers coated the total surface of the foam [Fig. 5 (a–b) and (d–e)].

However, their morphology shows clear differences, appearing quite smooth in the co-PPr case [Fig. 5 (f)], while it results much rough in the homo-PPr [Fig. 5 (c)]. The morphologies of the two devices can be attributed to the different microstructure as well as MMs (see Table 1) of the two types of macromolecules involved. Higher MMs values, together with the presence of 1,20-di(biphenoxide-A) eicosane moieties along the chains, could confer to the co-PPr the ability to better coat the substrate due to its higher viscosity. In this regard, in Fig. 5 (e) it is possible to appreciate that two separate parts of GF remained linked by polymeric co-PPr film formed after solvent evaporation. The experimental data reported above indicate the presence of an intimate contact at the interface GF/PPrs. This evidence suggested the existence of non-covalent interactions among PPrs and GF, able to accomplish the charge transfer process between the photoactive polymer and graphene substrate. At the same time, an uniform coating of polymer material was obtained, helping to protect the graphene surface from the direct exposure of light and OH^•^ radicals.

To finely explore the morphologies and appreciate the roughness parameters of porphyrins and cyclic-porphyrin polymers onto the graphene surface, we performed AFM analysis onto 2D reference nickel/graphene substrates. These samples were prepared following the same procedure used for 3D GF/cyclic porphyrin polymers. Lower magnification topographic AFM images (Fig. 3s ESI^†^) evidenced a well distributed and extended coating for homo- and co-PPrs films, if compared to porphyrin monomers. Nonetheless, higher magnification AFM analyses of the three samples demonstrated relevant morphological differences.

**Figure 6:**
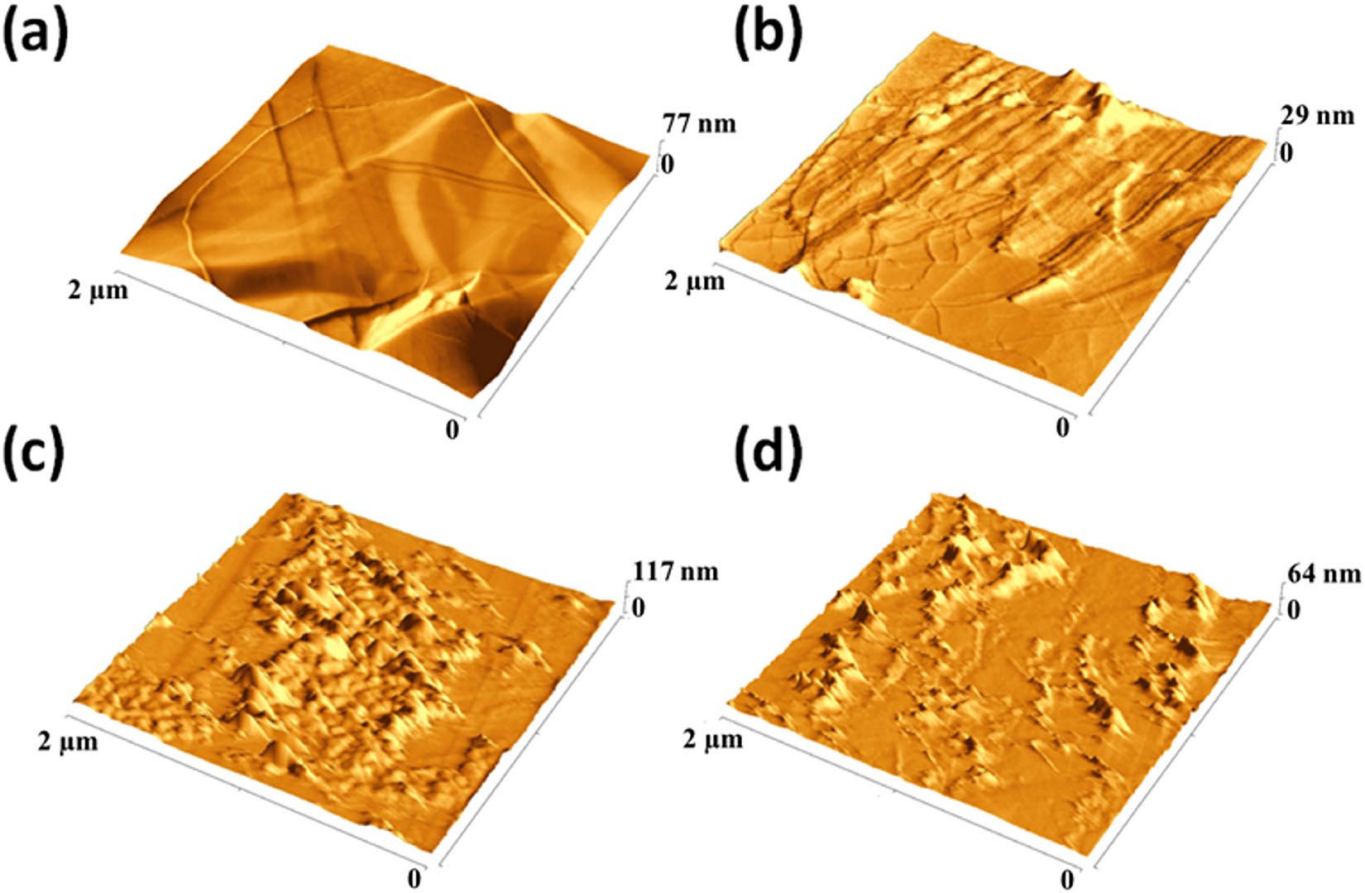
Topographic AFM images of (**a**) GF, (**b**) GF MPr, (**c**) GF homo-PPr and (**d**) GF co-PPr.

In Fig. 6 the AFM images at higher magnification collected for (a) pristine graphene surface, (b) graphene/porphyrin monomer, (c) homo-PPr and (d) co-PPr were reported. As it is possible to appreciate from Fig. 6(b), porphyrin monomers deposited by a DMF solution formed on the exposed surface round shape nano-aggregates as well as nano-tubular structures. The statistics on morphological parameters for this sample revealed a peak-to-peak (i.e. the highest probed height difference by the AFM tip) of 2 nm and a value of RMS of 2.3 nm (all rough surfaces exhibit perpendicular height fluctuations which are characterized by a Root Mean Square, RMS, quantifying the surface roughness). As the width of porphyrin units are estimated to be about 2 nm, we might suppose that they are tilted up perpendicularly to the surface forming non-photoactive H-nanoaggregates types. The round shaped nano-domains whose molecules, without specific form, possess a peak-to-peak of 19 nm. The homo-PPr and co-PPr AFM images [Fig. 6(c) and (d), respectively] show a better coverage of the underlying graphene and non-columnar structures are present. Nonetheless, more nano globular structures appear, with a peak to peak of 20–30 nm and a RMS of 9.2 nm for the homo-PPr, whereas 69 nm with a RMS of 4.7 nm were registered for the co-PPr. This difference can be attributed to the bigger sizes of macromolecules in the co-PPr materials than in homo-PPr [see gel permeation chromatography data (GPC), Table 1]. Moreover, the nanoaggregates on the surface of homo-PPrs appear very close each other [Fig. 6(c)], whereas the co-PPrs topography [Fig. 6(d)] clearly shows a much lower surface density of these nano globular shape onto the surface and, hence, a much higher flat surface available for photocatalytic processes. This fact can be attributed to the different cyclic hindered conformational geometries of the homo and co-polymers compared to the monomers. In particular, co-polymers are characterized by longer spacers between porphyrin units that further avoid the π-stacking between them and, as a consequence, strongly reduce the formation of non-active aggregates, as evident in Fig. 6(d).

### Photocatalytic activity

The photocatalytic activity under visible-light irradiation of the nano-hybrid materials was evaluated and compared by degrading MB dye water solution. As argued by Yan *et al*.^37^, MB could not be an appropriate probe molecule in photocatalytic test under visible light exposure since in this range it can give a contribution in photoabsorption. Thus, it can induce incorrect measurements of MB concentration during catalytic processes. We faced up this problem by measuring the absorption spectrum of aqueous MB solution (without the graphene/Ni foam) under visible light irradiation (see Fig. 6s ESI^†^) and subtracting these absorption contributions to the absorption profile produces by our systems during the exposure time. We underline that the use of MB is still considered an ISO procedure and hence its use allows to obtain an immediate and easy comparison of our results with those recently reported in literature by using similar procedures^13^.

**Figure 7:**
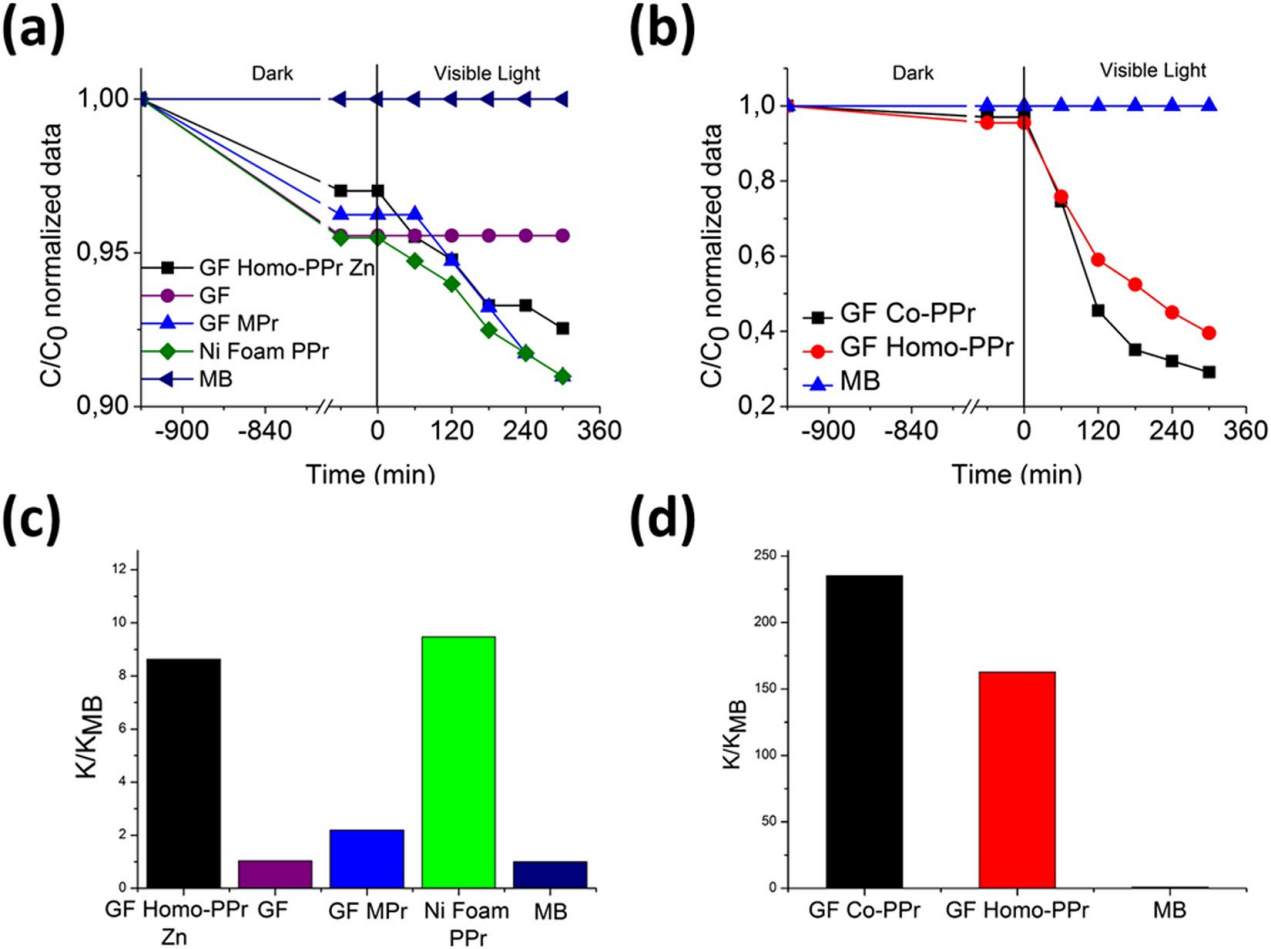
Photocatalytic activity of the (**a**) GF homo-PPr Zn (black line), GF (purple line), GF MPr (blue electric line), Ni Foam PPr (green line), and (**b**) GF Co-PPr (black line) and GF homo-PPr (red line) compared in both cases to the discoloration of pure MB under visible light irradiation. In (**c**) and (**d**) are reported the photocatalytic degradation efficiencies by using MB.

**Figure 8:**
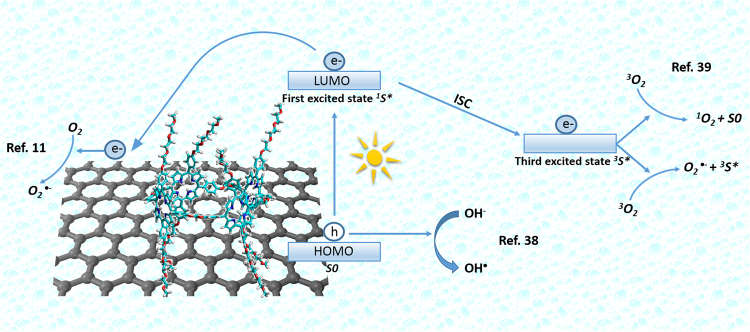
Proposed mechanism of the nanohybrid system GF/polyporphyrins for dye degradation in water.

As described in the experimental section, graphene polymer nanocomposites samples were immersed in 2 ml of MB solution 0.015 mM and left overnight in dark to reach the adsorption-desorption equilibrium. In Fig. 7 (a)-(b), the normalized C/C_0_ values (where C and C_0_ are the actual and starting MB concentrations, respectively) of MB as a function of the photo-exposure time during both the dark and visible-light experiments are reported. To dispel any doubts on the efficiency of the GF device in promoting photocatalysis, homo-PPrs were tested also on pristine Nickel foam surface (without graphene). As shown in Fig. 7 (a), homo-PPrs embedded on Nickel foam (Ni Foam homo-PPr, green trace) displayed 7% of decrement in C/C_0_ values, indicating a very low photocatalytic activity. Similarly, GF with porphyrin monomers, as well as GF/homo-PPr Zn did not give significant catalytic efficiencies [Fig. 7 (a)], whereas a huge increment in the degrading MB molecules was evident for the GF surface covered by the two different photoactive polymers [GF/homo and co-PPrs devices in Fig. 7 (b)]. Experimental data can be explained taking into account these behaviors of the electron-hole pairs formed under irradiation in the different systems. We demonstrated that the porphyrin monomers onto Ni foam did not show a significant activity, likely due to a too fast recombination of the electron-hole pairs formed by irradiation. When our porphyrins are coupled with graphene (porphyrins on graphene/Ni foam), the contact interface between them (i.e. the potential established across it) prevents charge recombination. In particular when our hybrid systems were irradiated by visible light, an electron was excited from the polyporphyrins ground state (S0) to first excited state (^1^S*), HOMO and LUMO band, respectively. After that, several pathways (see Fig. 8) are proposed^11,38–40^: (1) the photogenerated electrons in a first excited singlet state (^1^S*) are transferred towards graphene and oxygen dissolved in water is reduced in superoxide radical anion; (2) the first excited singlet state can decay to triplet excited state (^3^S*) by internal cross systems and thus transfer its energy to molecular oxygen (^3^O_2_) to generate singlet oxygen (^1^O_2_); (3) the molecular oxygen (^3^O_2_) can form superoxide radical anion by photon-induced electron transfer; (4) The excess of holes surviving in the porphyrin can migrate to the surface and react with OH^**−**^ to produce reactive oxygen species.

The electron transfer process and, as a consequence, photocatalytic activity of graphene/porphyrin composites, could be affected by morphological differences, as evidenced from SEM and AFM analyses of co-PPr, homo-PPr and porphyrin monomers (Figs 5 and 6). It is worth to note, indeed, that a typical dewetting phenomenon occurs during porphyrin monomer and polymers casting onto the graphene surface, forming porphyrin aggregates. The length of the spacers between porphyrin units can drive this phenomenon, which results characteristic for each polymer. As a consequence, changes in coating were observed. Moreover, the different coating of homo-PPr if compared to the co-PPrs (Fig. 3s ESI^††^) could derive also by their different molecular masse values. As far as the monomer case is concerned, we cannot exclude that its deficiency in photocatalytic activity on graphene surface could arise from an inadequate coverage of the underneath graphene substrate. Concerning the low photocatalytic activity of homo-PPr Zn, it might be ascribed to the discrete amount of unreacted monomer into the casted solution as well as its low degree of polymerization.

According to the Langmuir-Hinshelwood model, we also evaluated the photocatalytic reaction rate, k, given by the following equation: ln(C/C_0_) = −kt, where t is the time^41^. The values of the reaction rate k reported in the ordinate axis are normalized to the k value found in the absence of any catalyst materials (k/k_MB_). The data calculated for our samples were reported in the Fig. 7 (c)-(d), remarkably defining the followed order in term of PCE: GF/co-PPr > GF/homo-PPr ≫ Ni Foam PPr > GF-Pr-Zn > GF-Pr > GF. The higher performance of co-PPr than homo-PPr, could be attributed to the presence of secluded porphyrins along the cyclic chains that better prevent agglomeration. Outstanding, the amount of porphyrin polymers in co-PPrs was about 60% lesser than homo-PPrs. In any case, we want to highlight that 400 μg of polymers supported on 6 mg of GF are able to explicate outstanding photocatalytic efficiencies if compared with the recently findings^9,42–44^. We want to stress also that our device is a freestanding formulation, avoiding further separation step from water solution, needed in case of dispersed catalyst^42–44^. Obviously, different parameters related to the co-PPrs photoability have to be addressed, such as the length and structure of spacer between porphyrin units, the MM values and distribution as well as polymer solubility.

Regarding the practical water remediation issue, recyclability is an important property required by photocatalytic devices. Therefore, we performed some recyclability tests. In particular the stabilities of GF/homo-PPr and GF/co-PPr devices was evaluated performing MB degradation test for three times. After the first cycle, carried out as described in the experimental section, the photocatalysts were removed from water, washed thrice with water, dried for 12 hours and used for the second and the third cycles. The Fig. 5s (a) (see ESI^†^), concerning GF/homo-PPr, indicates about 60% degradation of MB in the first cycle while it decreases to about 40% and 20% in second and third cycle, respectively. Whereas, from the Fig. 5s (b), the percentage of MB degradation for GF/co-PPr decreases from 71% to the 22% from the first to the third cycle. Considering that each run lasts 300 minutes, our systems are able to perform an outstanding photoactive action up to 700 min.

**Figure 9:**
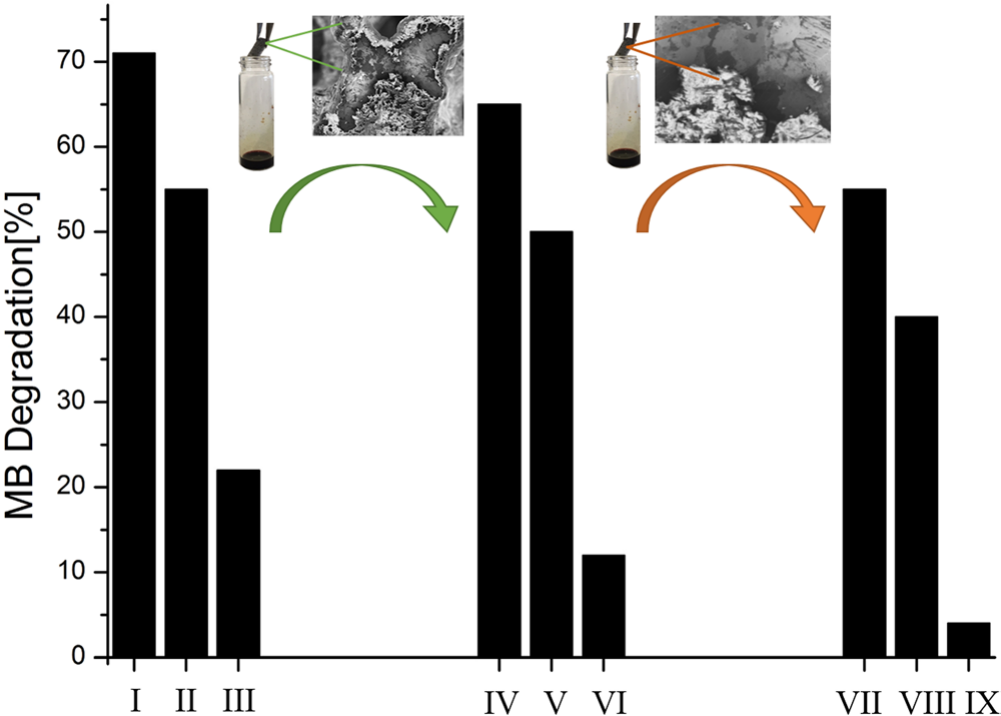
Recyclability after re-deposition of co-PPr on degradated GF/co-PPr.

SEM analysis of GF/homo-PPr and GF/co-PPr [Fig. 5s (c,d) ESI^†^], carried out after the third run, shows a sensible damage of polymer coating on graphene surface. This phenomenon could be attributed to the reactive oxygen species (ROS) which have acted a significant erosion. This evidence suggests that the reduction of photocatalytic action was mainly due to degradation of macromolecules on graphene surface. To test if graphene surface was still able to exploit its co-catalytic properties, we embedded again the GF with the co-PPrs polymers solution, washed with DMF and dried for 12 hours obtaining a restored material for a new recyclability experiment. The same procedure was repeated two times. Figure 9 reports the % of MB degradation for each experiment, evidencing that device performance was kept almost constant in the second experiment with a partial decrease in the third one.

## Conclusion

The combination of organic photosensitizers with graphene-based materials has become a hot topic in water remediation. We demonstrate a novel innovative approach that consists in combining properties of graphene 3D and porphyrin polymers in a freestanding device. This simple assembly method promotes a direct and extensive contact between the graphene and the photosensitizer polymers through the instauration of non-covalent interactions at the materials interface. At the same time, the polymer coating can confine graphene materials to the direct exposure of light, dyes and OH^•^ radicals, which drastically reduce the photocatalytic efficiencies. On the other hand, the use of sterically hindered cyclic polyporphyrins increases the number of photoactive sites, limiting the formation of agglomerates that hampered the desired charge transfer process onto the GF surface. The photocatalytic degradation tests carried out by using MB in water solution showed higher efficiencies of homo- and co-polyporphyrins compared to porphyrin monomers coating. Additionally we highlighted that only 400 μg of polymers supported on 6 mg of GF explicated an outstanding photocatalytic efficiencies with a good recyclability capabilities. This evidence can be a game changer in the formulation of polyporphyrins as photoactive compounds for such kind of graphene hybrid materials. The co-monomers nature, the MM values, the sequence length as well as the topology of the co-polymers, could significantly influence the photoactivity behaviour of cyclic polyporphyrins materials, reserving new stimulating finding. At the same time, the polymer deposition methods and parameters could be also taken into account to control aggregate formation. As for Ni/GF, interesting future perspectives put forward the use of nickel free graphene 3D as charge transfer material.

## Methods

### **Instruments and measurements**

#### Gel Permeation Chromatography (GPC)

A Waters 515 HPLC pump, connected to four Ultra-Styragel HR columns joined in series (in the order: HR4, HR3, HR2 and HR1), and a Waters R401 differential refractometer, were used for GPC analysis. Polymer solutions (1 mg/ml, in THF) were injected and eluted at a flow rate of 1 ml/min. Calibration curve was obtained using a set of primary polymethylmethacrylate (PMMA) standards.

#### Matrix Assisted Laser Desorption Ionization–Time Of Flight (MALDI-TOF)

MALDI /mass spectra were recorded in reflector mode using a 4800 MALDI TOF/TOF™ Analyzer (Applied Biosystem, Framingham, MA, USA), equipped with a Nd:YAG laser (λ = 355 nm) and working in positive-ion mode. External calibration was performed using an Applied Biosystems calibration mixture consisting of polypeptides with different molecular weight values. Mass accuracy was about 50 ppm. The samples were prepared by mixing approximately 0.1 mmol of the monomer or polymer and 40 mmol of trans-3-indoleacrylic acid (IAA, used as a matrix) on the sampler target, using THF as a solvent. The m/z nominal values reported in the spectra, are referred to molecular ions constituted by the most abundant isotopes of each element present in the molecule.

#### UV-Vis spectroscopy

UV-Visible spectra were recorded on a Shimadzu spectrophotometer at room temperature using toluene as a solvent and quartz cuvettes with a path length of 1 cm. The porphyrin content in co-PPr was evaluated by considering the absorption value at 423 nm (λmax for porphyrin group) using Beer’s law and a molar absorption ε of 378692, determined for porphyrin monomer in toluene. The calculated compositions were reported in Table 1.

#### Atomic Force Microscopy (AFM)

AFM analyses were performed using a Bruker-Innova microscope operating in high-amplitude mode and ultra-sharpened Si tips were used (MSNL-10 from Bruker Instruments, with anisotropic geometry, radius of curvature ∼2 nm, tip height ∼2.5 μm, front angle ∼15°, back angle ∼25°, side angle 22.5°). The Si tips were substituted as soon as a resolution loose was observed during the AFM images acquisition. The AFM images were analyzed by using the SPMLABANALYSES V7.00 software. In order to give representative parameters, statistics on the AFM images were carried out over different areas of the same sample.

#### XPS analysis

XPS were performed by a PHI ESCA/SAM 5600 Multy technique spectrometer with the use of an Mg standard X-ray source. During the analyses, the pressure in the chamber was ~10^−9^ Torr. The measurements were carried out at 45° photoelectron take-off angle relative to the sample surface with an acceptance angle of ±3°. The analyzer pass energy was set at 23.5 eV for the high-resolution spectra. The binding energy (BE) scale was calibrated by centering the C1s signal of the aliphatic/aromatic component at 285.0 eV). The analyses were performed on 2 mm diameter surface area of the sample, much greater than the lateral size of any carbon island on the nickel foam surface.

#### Raman spectroscopy

Micro-Raman Stokes spectra were taken in backscattering geometry with a HORIBA Jobin-Yvon system, equipped with Olympus BX41 microscope. He-Ne laser radiation at a wavelength of 632.8 nm is focused to a spot size of about 1 μm by a 100× objective. The laser power on the sample was about 5 mW, and a 550 mm focal length spectrometer with 1800 lines/mm grating was used.

#### SEM analysis

The morphology of hybrid structure were analyzed by scanning electron microscopy (SEM, Gemini 152 field emission SEM Supra 25, Carl Zeiss, Oberkochen, Germany) using a Zeiss Supra 25 microscope operating at 2.0 kV, both in the Inlens and secondary lens operation mode.

#### Materials and Reagents

All solvents and basic materials were commercial products appropriately purified before use.

### **Synthetic procedures**

#### Synthesis of porphyrin homopolymers (homo-PPrs)

The homopolyformal [homo-PPr in Fig. 2 (a)] was synthesized according to the method described elsewhere^23^ by reaction of 5,10-di[p-(9-methoxytriethylenenoxy)phenyl]−15,20-di[p-hydroxyphenyl]-porphyrin (see ESI^†^ for synthesis method) with CH_2_Br_2_. In a 2 mL vial, equipped with magnetic stirrer and placed in a water bath at 70 °C, 10 mg (0.01 mmol) of 5,10-di[p-(9-methoxytriethylenenoxy)phenyl]−15,20-di[p-hydroxyphenyl]-porphyrin were solubilized in 0.5 mL of N-methylpirrolidinone (NMP) together with 3 mg (a large molar excess) of powdered KOH. After 1 hour, 0.3 mL of CH_2_Br_2_ (a large excess) were added and the vial closed. After 24 hours, the solution was poured into 5 mL of 5% HCl under vigorous stirring and the obtained suspension was centrifuged to give a purple polymeric residue. The material was solubilized in 2 mL of THF, precipitated in 5 mL of water, centrifuged and dried in vacuum for 24 hours (yield about 80%). The polymer was characterized by MALDI-TOF mass spectrometry, GPC and UV-Vis spectroscopy. Homo-polymer based on zinc-porphyrin monomer was synthesized in a similar way starting from the specific porphyrin derivative.

#### Synthesis of porphyrin copolymer (co-PPr)

Random copolymer [co-PPr in Fig. 2 (b)] was synthesized by interfacial etherification reaction. According to the method described elsewhere^22^, tetrabutylammonium bromide (TBAB) as the phase-transfer agent and a 50% (in moles) mixture of monomers (see ESI^†^ for synthesis method) were used, in the presence of a large excess of dibromomethane. Typically, 11 mg of 5,10-di[p-(9-methoxytriethylenenoxy)phenyl]−15,20-di[p-hydroxyphenyl]-porphyrin (0.011 mmol) and 8.1 mg of di(bisphenoxy-A)eicosane (0.011 mmol) were solubilised in 3 mL of toluene in a 10 mL closed vial and heated at 70 °C. Then, 4 mg of NaOH (0.1 mmol, a large molar excess), solubilised in 1 mL of water together with 7.1 mg of TBAB (0.022 mmol), were added and the solution was stirred at the same temperature for 1 hour before adding 0.5 mL of CH_2_Br_2_. After 24 hours, the organic solution was separated from the aqueous one and poured, under stirring, into a solution of 10 mL of EtOH acidified by 0.5 mL of CH_3_COOH. After separation by centrifugation, the precipitated material was dissolved in THF and precipitated in EtOH a further two times, washed with H_2_O, then dried under vacuum. 14 mg of a purple fibrous polymeric material was recovered with a yield of about 75%. The copolymer was characterized by MALDI-TOF mass spectrometry, GPC and UV-Vis spectroscopy.

#### Synthesis of GF

Nickel foams (GoodFellow, 0.45 g/cm^2^, porosity 95%, 20 pores/cm, purity 95% and 1.6 mm thickness) were used as 3D scaffold templates for the CVD growth of GF. They were cut into pieces of 5 × 10 mm, then immersed in acetic acid to remove Ni native surface oxide layer and finally placed in the chamber of the AIXTRON’s Black Magic Chemical Vapor Deposition (CVD) system. The chamber was firstly pumped down to 4 × 10–3 mbar and then gradually heated both from the top and bottom side of the chamber up to 900 °C and maintained at this temperature for 20 minutes under Ar and H_2_ flows (600 and 400 sccm, respectively) to further clean the surface and eliminate the residual thin surface oxide layer. Then, the temperature was raised up to 1000 °C and the CH_4_ flow was opened for 40 minutes (flow rates of 20, 600 and 1000 sccm for CH_4_, Ar and H_2_, respectively) and then cooled down at 15 °C/min to grow the graphene layers on the Ni foam surface. The working chamber pressure was maintained 25 mbar both in the cleaning and growth steps.

#### Hybrid nanocomposites preparation

About 10 mg of each sample (monomer, homopolymers and copolymer) was dissolved in 1 mL of dimethylformamide (DMF). After, a GF piece of 5 mm × 20 mm was immersed in each polymeric solution. Samples were statically impregnated in the mixture overnight, then removed from vials. The resulting hybrid materials were dried under vacuum overnight at 50 °C.

#### Photocatalytic activity

The photocatalytic activities of the nanohybrids were evaluated through the degradation of MB dye under visible light irradiation. A xenon lamp operating at 1.5 mW/cm^2^ and equipped with a cut-off filter (λ > 400 nm) was used as the light source. For each experiment, a MB dye aqueous solution (0.015 mM) was prepared. The samples were immersed in 2 ml of the MB solution and left for 12 h in the dark to reach the adsorption-desorption equilibrium. Afterwards, at given time intervals of 1 h, the variation of the concentration of the MB was spectrophotometrically evaluated using a PerkinElmer Lambda 45 UV–Vis spectrophotometer, observing the absorption peaks at 664 nm. The photodegradation ratio was defined as C/C_0_ where C is the absorption of the dye at a certain time and C_0_ represents the absorption value of the MB initial concentration. In particular, these curves were previously subtracted by the contributions of MB photoabsorption, that were measured by irradiating MB aqueous solution (without graphene/Ni).

## Electronic supplementary material

Supplementary information
