# Supplementary material for: Freestanding photocatalytic materials based on 3D graphene and polyporphyrins
Source: Sci Rep. 2018 Mar 22;8:5001. doi: 10.1038/s41598-018-23345-y (PMC5864880; doi:10.1038/s41598-018-23345-y)
Supplement: Supplementary file 1 — Supplementary information [file 41598_2018_23345_MOESM1_ESM.docx]

**Supporting Information for:**

Freestanding photocatalytic materials based on 3D graphene and polyporphyrins

Martina Ussia, Elena Bruno, Emanuela Spina, Daniele Vitalini, Giovanna Pellegrino, Francesco Ruffino, Vittorio Privitera, Sabrina C. Carroccio*

*SI correspondence to:

Sabrina Carola Carroccio: sabrinacarola.carroccio@cnr.it

Synthetic procedure of monomers

Synthesis of 5,10-di[p-(9-methoxytriethylenenoxy)phenyl]-15,20-di[p-hydroxyphenyl]-porphyrin monomer. The monomer was synthesized by reaction between tetrakis-p(hydroxyphenyl)-porphyrin and 9-methyltriethyleneoxy chloride in aqueous alkaline solution, as reported elsewhere.^23^

Synthesis of 5,10-di[p-(9-methoxytriethylenenoxy)phenyl]-15,20-di[p-hydroxyphenyl]-zinc porphyrin monomer.

Zinc 5,10-di[p-(9-methoxytriethylenenoxy)phenyl]-15,20-di[p-hydroxyphenyl]-porphyrin complex was obtained, as previously reported, by reaction between the 5,10-di[p-(9 methoxytriethylenenoxy)phenyl]-15,20-di[p-hydroxyphenyl] porphyrin and the teen excesses fold of zinc acetate in pyridine.^24^

Synthesis of 1,20-di(bisphenoxy-A)eicosane monomer. 1,20-di(bisphenoxy-A)eicosane was obtained from 1,20-dibromo-eicosane and bisphenol-A according to the method mentioned elsewhere.^25^

**MALDI-TOF analysis of homo-PPr and co-PPr**


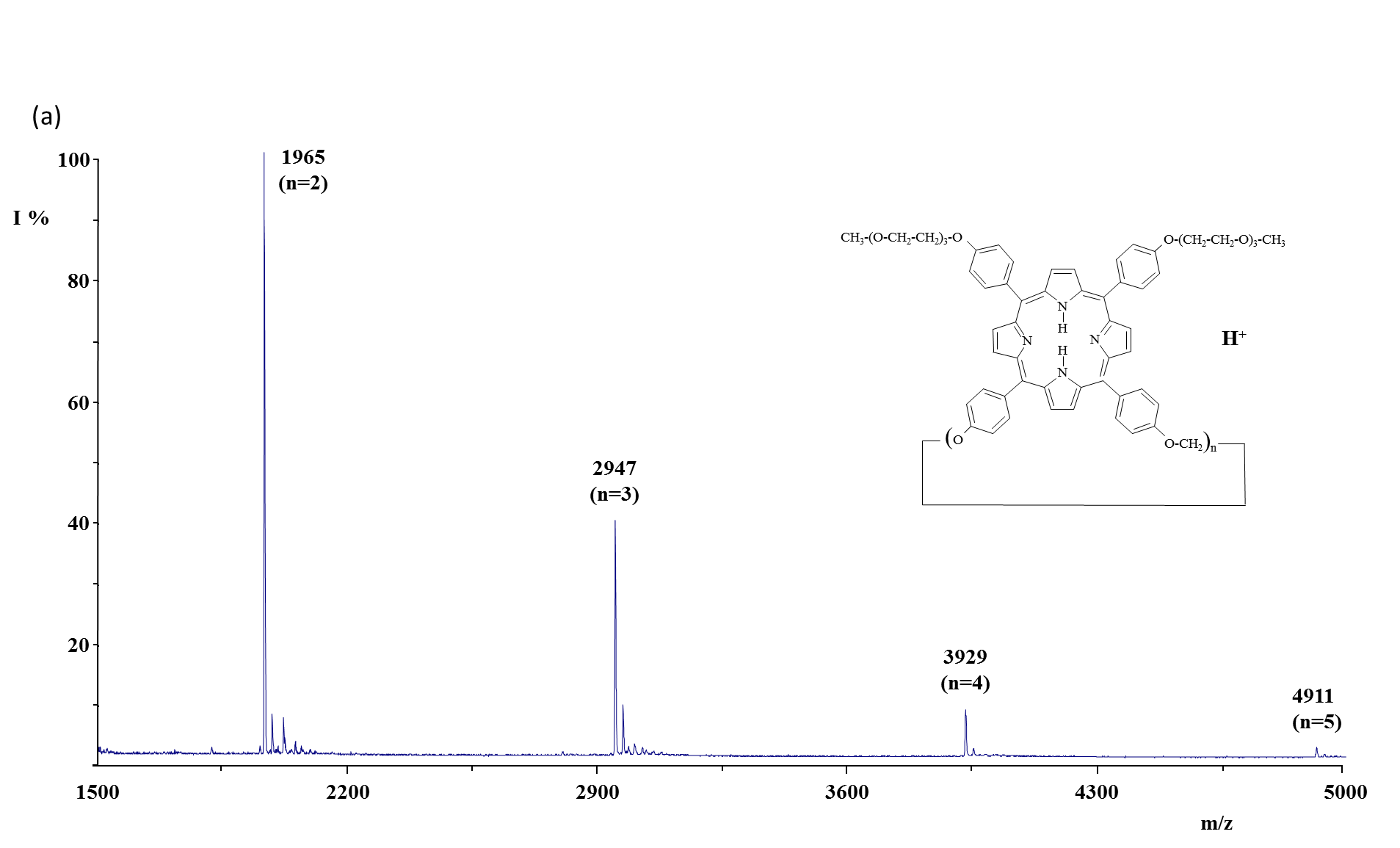


**
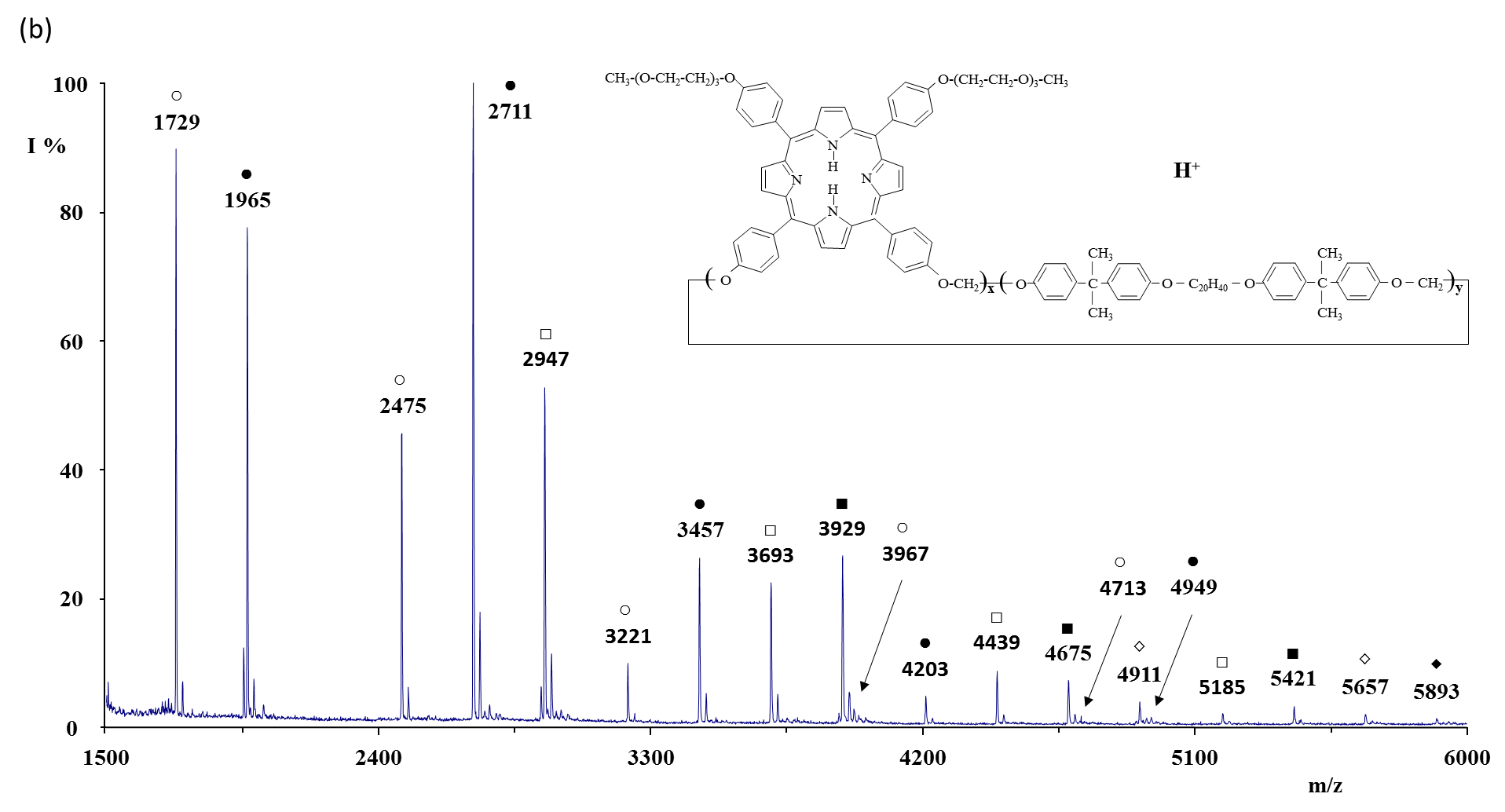
**

**Figure 1s (a)-(b).** Positive MALDI-TOF mass spectrum, acquired in reflection mode, of **(a)** homo-PPr and **(b)** co-PPr (for the structural assignments see **Table 1s**).

In **Figure 1s (a)** is reported the MALDI TOF spectrum of homo-PPr, acquired in positive and reflectron mode, in the mass range *m/z* 1500-5000. From the inspection of the registered MS spectrum, it is possible to notice the appearance of a series of prominent peaks assigned to protonated adducts of cyclic PPrs oligomers. In **Figure 1s b** is showed the MALDI mass spectrum of co-PPr in the mass *m/z* range 1500-6000. Peaks appearing in the **Figure 1s b** were identified, and correspond to different families of protonated cyclic adducts of cyclic PPrs oligomers. Peaks detected, indicated the presence of oligomers up to pentamers, although higher oligomers could be formed and related peaks lost in the background.

| **X**    **Y** | **1**  **○** | **2**  **●** | **3**  **□** | **4**  **■** | **5**    **◇** | **6**    **◆** |
| --- | --- | --- | --- | --- | --- | --- |
| **0** |  | **1964** | **2946** | **3928** | **4910** | **5892** |
| **1** | **1728** | **2710** | **3692** | **4674** | **5656** |  |
| **2** | **2474** | **3456** | **4438** | **5420** |  |  |
| **3** | **3220** | **4202** | **5184** |  |  |  |
| **4** | **3966** | **4948** | **5930** |  |  |  |
| **5** | **4712** | **5694** |  |  |  |  |

**Table 1s.** Molecular mass of the cyclic compounds present in co-PPr, detected in MALDI TOF mass spectrum as M-H^+^. Symbols reported under x numbers refer to families of cyclic oligomers containing the same number of porphyrin units, detected in the mass spectrum of Fig. 1s a-b.

**
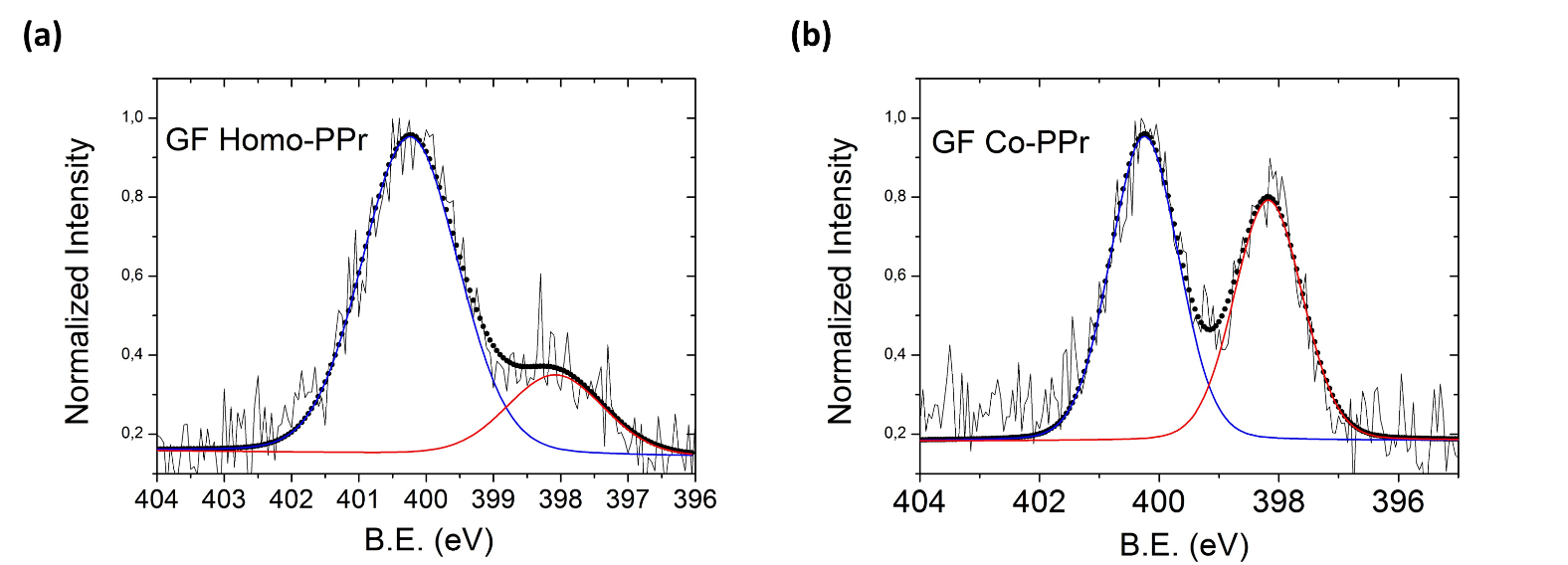
XPS analysis of homo- and co-PPr powders**

**
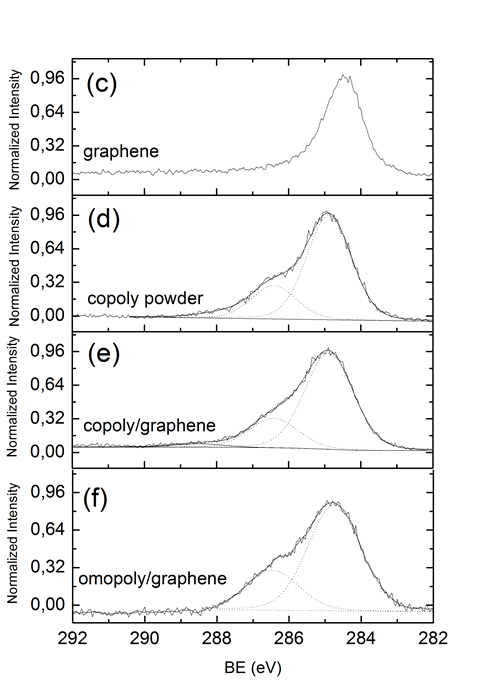
**

**Figure 2s.** (a)-(b) N1s XPS spectra carried out on the powders of (a) homo-PPr and (b) co-PPr; (c)-(f) C1s XPS spectra of (c) graphene (d) co-PPr powder (e) GF Co-PPr and (f) GF homo-PPr

The C1s XPS region, as detected for (c) graphene, (d) co-polymer powder, (e) co-polymer and (f) omo-polymer adsorbed on graphene, is shown in figure 2s. In the case of the graphene substrate, the C1s signal consists of a narrow band (FWHM of 1.2 eV) centered at 284.7 eV. The C1s XPS spectrum obtained from the powder of the cyclic poliporphyrin co-polymer (fig 2s-d) reveals the presence of two components positioned at 285.0 eV and at 286.5 eV, both with FWHM of 1.5 eV. Such bands are due, respectively, to the C-C, C=C , C-H and to the C-O,C=N,C-N species of the porphyrin structure **^35^**.The spectra related to the two polyporphyrin compounds adsorbed on graphene [**fig.s 2s (e) and 2s(f)**] are very similar to that of the powder. Nevertheless, a slight enlargement of the FWHM is observed after the surface functionalization (1.7 eV vs 1.5 eV), as an effect of the interaction of the molecule with the surface **^28,29^**.

**AFM analysis of GF/homo-PPr and GF/co-PPr**


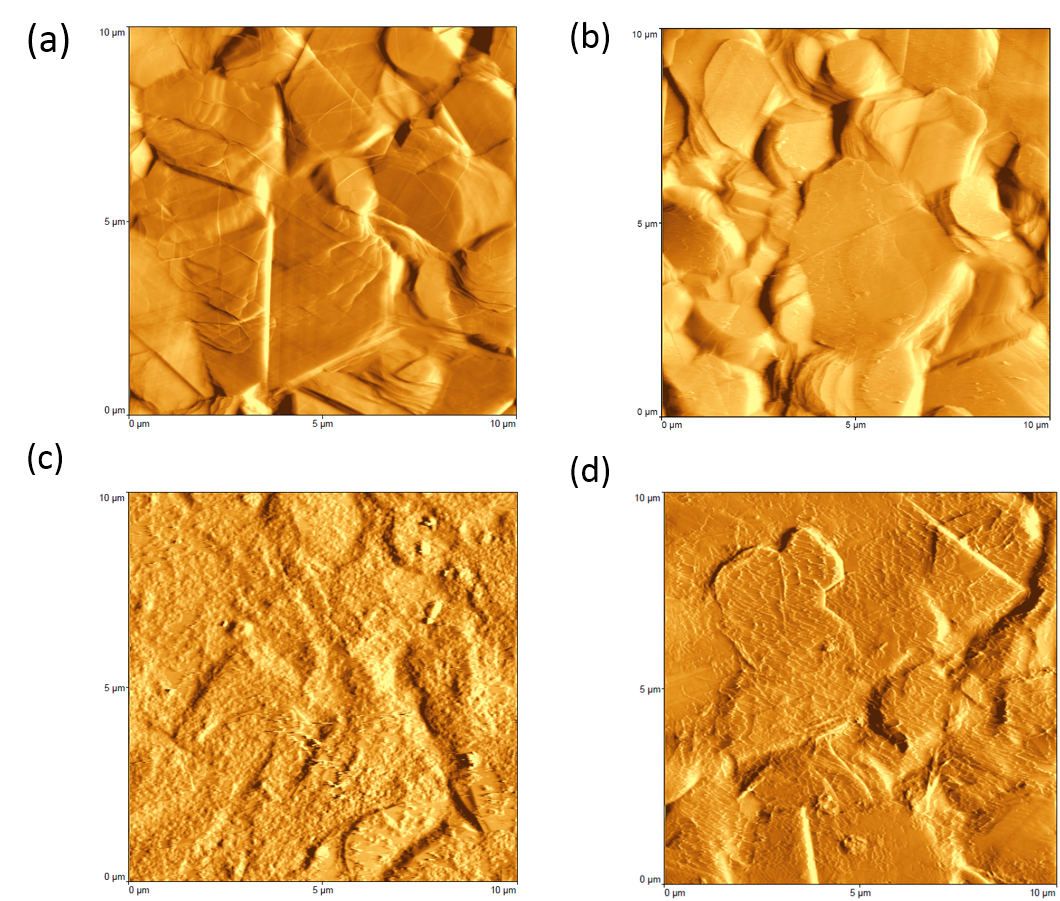


**Figura 3s.** Topographic AFM image at lower magnification of (a) GF, (b) GF MPr, (c) GF homo-PPr and (d) GF co-PPr.


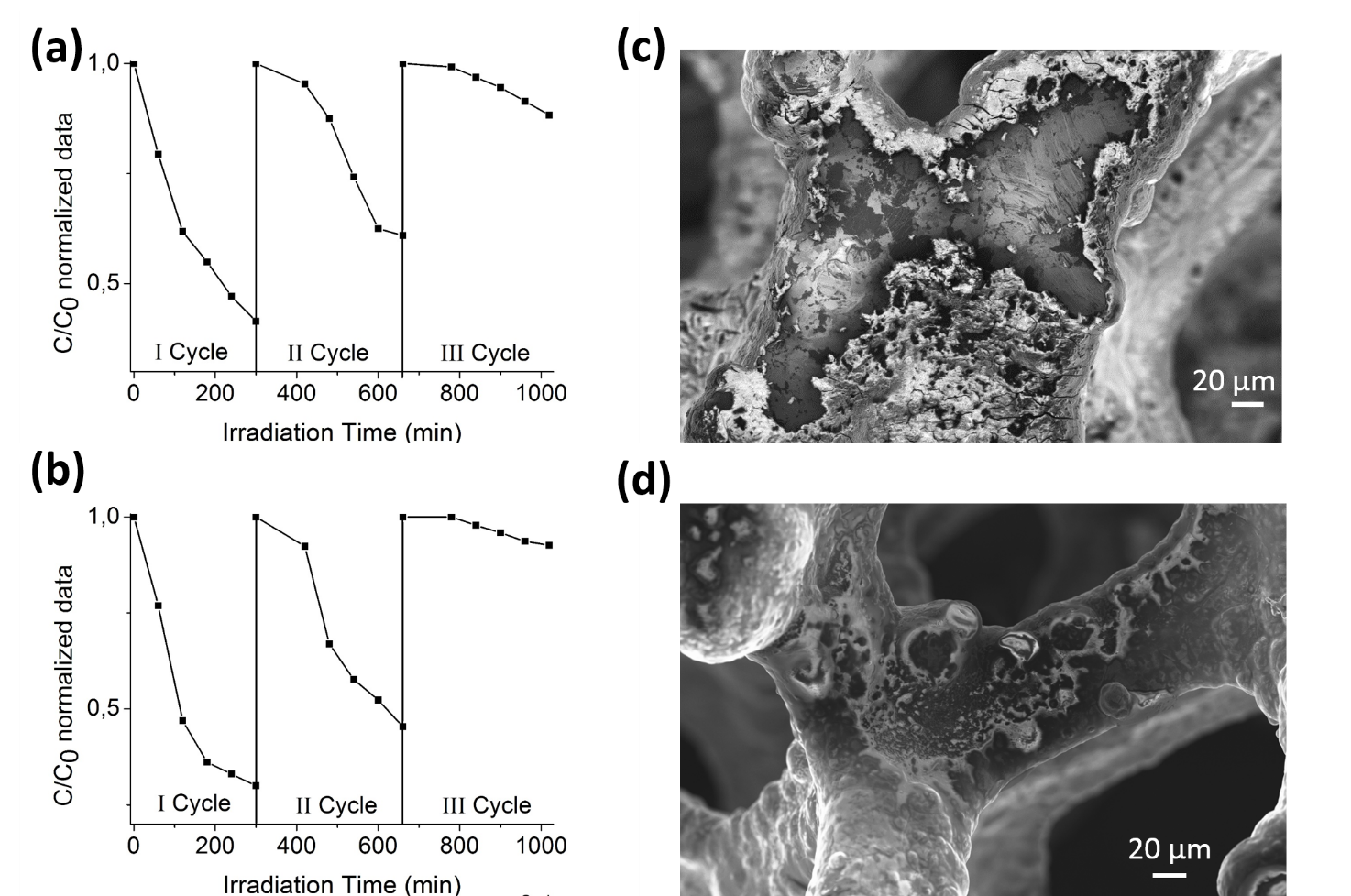


**Figure 4s.** Recyclability of (a) GF/Homo-PPr and (b) GF/Co-PPr for three subsequent cycles. SEM images obtained in the Inlens mode at 1.00 KX magnification of (c) GF Homo-PPr and (d) GF Co-PPr after the third cycle


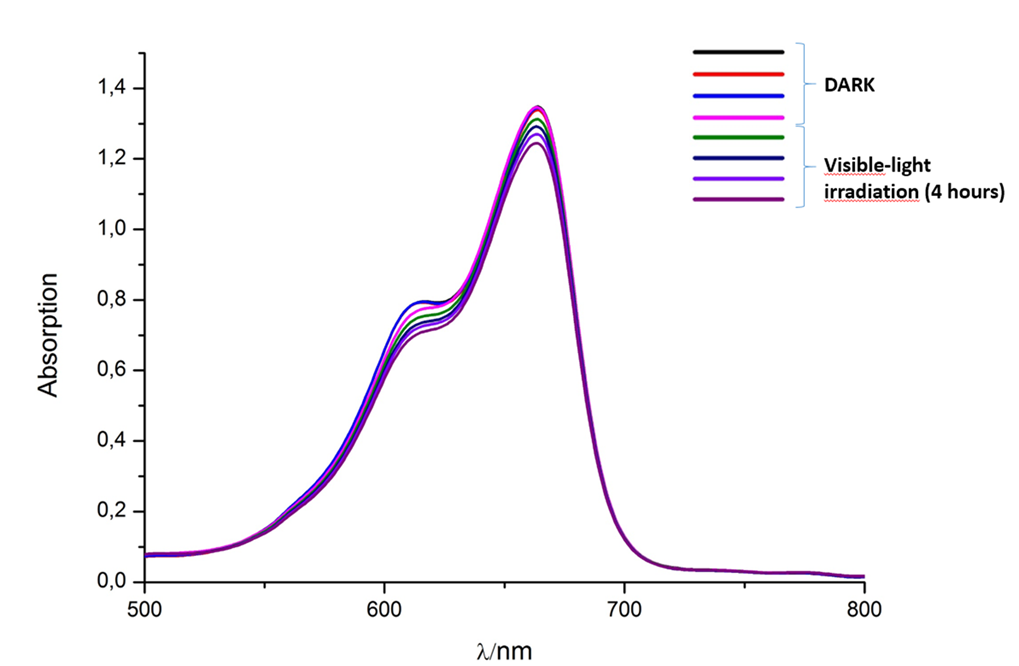


**Figure 5s.** Absorption spectra of MB solutions, registered in dark and under VL irradiation up to 4 hours
